# An update of predictive biomarkers related to WEE1 inhibition in cancer therapy

**DOI:** 10.1007/s00432-023-05527-y

**Published:** 2024-01-17

**Authors:** Zizhuo Wang, Wenting Li, Fuxia Li, Rourou Xiao

**Affiliations:** 1 Department of Gynecology and Obstetrics, Tongji Hospital, Tongji Medical College, Huazhong University of Science and Technology, Wuhan, 430030 People’s Republic of China; 2 Department of Gynecology, First Affiliated Hospital, Shihezi University, Shihezi, 832000 Xinjiang People’s Republic of China; 3 Department of Obstetrics and Gynecology, Zhongnan Hospital of Wuhan University, Wuhan, 430071 Hubei People’s Republic of China

**Keywords:** Biomarkers, Cancer therapy, WEE1 inhibition

## Abstract

**Purpose:**

WEE1 is a crucial kinase involved in the regulation of G2/M checkpoint within the cell cycle. This article aims to comprehensively review the existing knowledge on the implication of WEE1 as a therapeutic target in tumor progression and drug resistance. Furthermore, we summarize the current predictive biomarkers employed to treat cancer with WEE1 inhibitors.

**Methods:**

A systematic review of the literature was conducted to analyze the association between WEE1 inhibition and cancer progression, including tumor advancement and drug resistance. Special attention was paid to the identification and utilization of predictive biomarkers related to therapeutic response to WEE1 inhibitors.

**Results:**

The review highlights the intricate involvement of WEE1 in tumor progression and drug resistance. It synthesizes the current knowledge on predictive biomarkers employed in WEE1 inhibitor treatments, offering insights into their prognostic significance. Notably, the article elucidates the potential for precision medicine by understanding these biomarkers in the context of tumor treatment outcomes.

**Conclusion:**

WEE1 plays a pivotal role in tumor progression and is a promising therapeutic target. Distinguishing patients that would benefit from WEE1 inhibition will be a major direction of future research.

## Introduction

The emergence of molecular signature-based targeted therapy has revolutionized the field of antitumor treatment (Lee et al. 2018). In particular, the investigation of the DNA damage and repair pathway has garnered significant attention in recent years. Notably, numerous clinical trials have provided evidence that WEE1 inhibitors exhibit encouraging efficacy. Furthermore, when combined with other therapies, WEE1 inhibition can lead to synergistic anti-tumor effects in several cancer types with limited treatment alternatives, such as recurrent uterine serous cancer with *TP53* mutation (Liu et al. 2021), high-grade serous ovarian cancer with platinum resistance (Leijen et al. 2016b; Lheureux et al. 2021), and unresectable pancreatic cancer (Cuneo et al. 2019). Despite these encouraging advances, the beneficiaries of WEE1 inhibition remain uncertain. In other words, development of predictive biomarkers and identification of the appropriate population that would respond to WEE1 inhibitors are imperative. To this end, this article aims to provide a comprehensive review of the existing literature on the association the role of WEE1 as a therapeutic target in tumor progression and drug resistance. We also summarize the current progress on the predictive biomarkers employed in the treatment of tumors targeting WEE1.

## The mechanism of action of WEE1 inhibitors

**Fig. 1:**
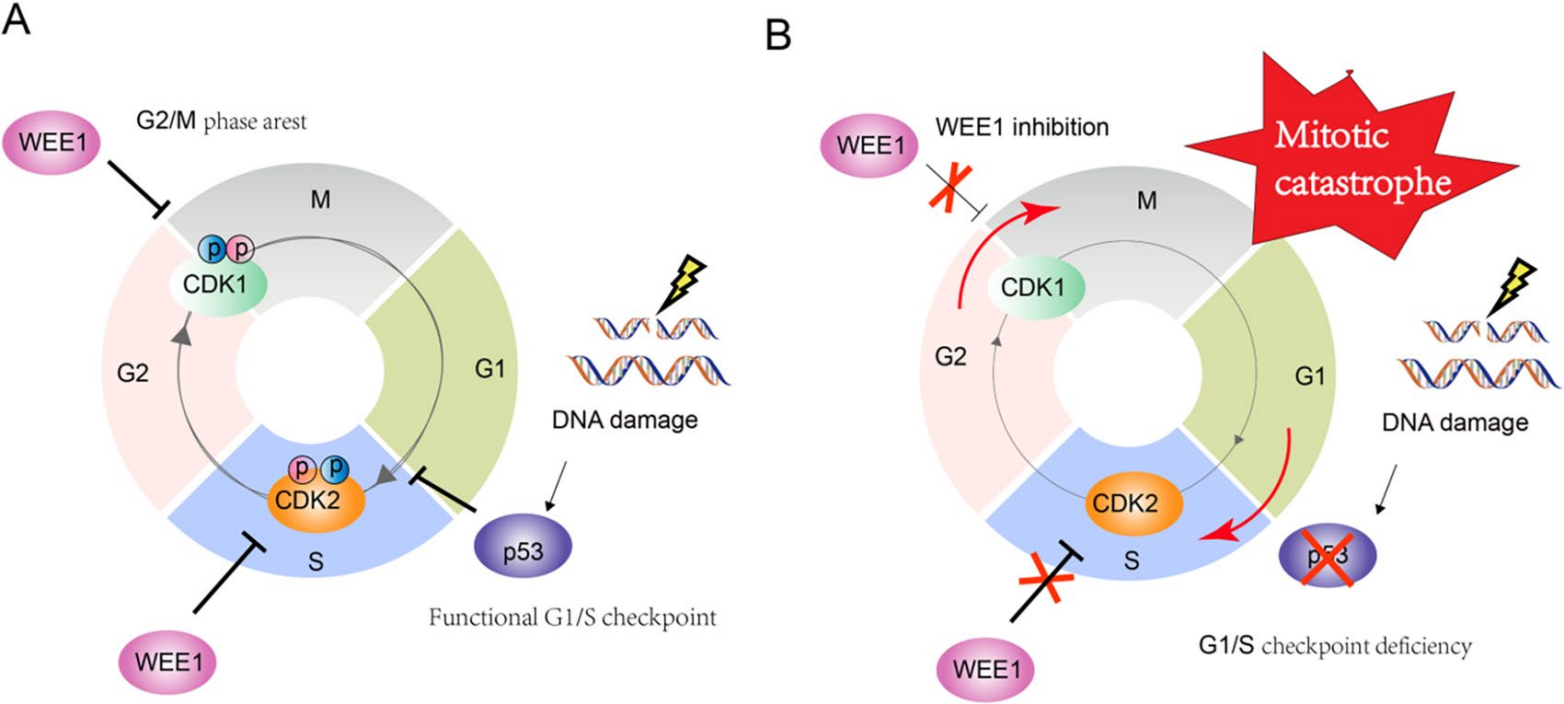
Cell cycle checkpoint regulation mechanism. **A** Cell cycle regulation in cells with intact cell cycle checkpoint function when DNA damage occurs; **B** Cell cycle regulation after G1/S checkpoint deficiency combined with WEE1 inhibition

Cell cycle regulation plays a vital role in maintaining DNA integrity (Campos and Clemente-Blanco 2020), which, to a great extent, depends on the cell cycle checkpoints, namely the G1/S and G2/M checkpoints (Poon 2016). The p53 protein (encoded by the *TP53* gene) plays a crucial role in DNA damage response by suppressing the G1/S cell cycle checkpoint (Jackson and Bartek 2009). The other pivotal checkpoint is the G2/M checkpoint, which is primarily regulated by WEE1 kinase (Smith et al. 2020). In the event of additional DNA damage during replication or failure to repair the initial damage, the WEE1 kinase hinders the progression of cell cycle into M phase by phosphorylating and inhibiting the activity of cyclin-dependent kinases CDK1/2 (Russell and Nurse 1987). This results in effective extension in the duration of DNA repair (Fig. 1A).

Unfortunately, in the context of human cancers, *TP53* is the most frequently mutated tumor suppressor across various cancer types, with high-grade serous ovarian cancer exhibiting the highest mutation rate (Zehir et al. 2017). Despite the notorious role of *TP53* mutations in cancer, pharmacological intervention specifically targeting p53 mutations has been limited, with only a few targeted drugs, such as PRIMA-1 and PRIMA-1Met (APR-246), being tested in early clinical trials. In *TP53*-mutant tumor cells, the WEE1 checkpoint encounters heightened pressure and thus presents a potential target for therapeutic intervention (Smith et al. 2020). Inhibition of WEE1 facilitates or even expedites mitotic progression, leading to an increase in genomic instability (Matheson et al. 2016), which ultimately result in mitotic catastrophe (Matheson et al. 2016) (Fig. 1B). Besides, WEE1 also regulates CDK2 during the S phase to stabilize the replication machinery (Beck et al. 2012; Li et al. 2020a). Hence, the coordination of diverse cellular division events by WEE1 undeniably establishes its significance as a potential target for clinical utilization in the treatment of tumors (Fig. 1B).

## WEE1 and cancer

### WEE1 expression in tumors

Recent studies have demonstrated that up-regulation of WEE1 is prevalent in various types of tumors (Beck et al. 2012), particularly in those exhibiting loss of p53 function (De Witt Hamer et al. 2011; Lau and Pardee 1982). For instance, a comprehensive comparison of 34 cancer-versus-normal data sets, revealed increased expression of WEE1 mRNA in 77% of the samples (Mir et al. 2010). Similarly, heightened expression or increased activity of WEE1 was observed in glioma (Mueller et al. 2014; Music et al. 2016), liver cancer models (Masaki et al. 2000, 2003), medulloblastoma (Harris et al. 2014), seminoma (Mir et al. 2010), breast cancer, and osteosarcoma (PosthumaDeBoer et al. 2011; Wang et al. 2011). Collectively, these aforementioned studies suggest that WEE1 level is elevated in human cancer and thus has the potential to serve as a molecular marker for tumors.

### The role of WEE1 in tumor progression

Inhibiting WEE1 effectively impeded the proliferation and migration of colorectal cancer liver metastases endothelial cells (CLMECs), leading to impaired vascular endothelial formation (Webster et al. 2017). Furthermore, WEE1 expression was closely associated with tumor-free survival rate, tumor burden, and the incidence of ulcer in melanoma (Magnussen et al. 2012), while WEE1 repression significantly reduced melanoma metastasis (DiSano et al. 2019). Additionally, the study by Magnussen et al. revealed a gradual increase in WEE1 expression during the process of carcinogenesis, with the highest expression observed in patients who had developed tumor metastasis (Magnussen et al. 2012). Furthermore, inhibiting WEE1 expression in glioma cells resulted in cell death (Sancar et al. 2004). These findings collectively suggest that WEE1 may promote tumor progression.

### The role of WEE1 in drug resistance

DNA repair mechanisms are considered as a significant contributor to resistance against DNA damage therapies (Dibitetto et al. 2022). WEE1 suppression could overcome resistance to EGFR-TKIs inhibitors and enhance the efficacy of cisplatin and gemcitabine (Liu et al. 2019). Additionally, Li et al. (2020b) demonstrated the significant impact of the DGKA-c-Jun-WEE1 signaling axis on platinum sensitivity in platinum-resistant ovarian cancer cells. A study involving 287 patients with advanced high-grade serous ovarian cancer observed higher expression of WEE1 in samples that recurred after initial chemotherapy compared to pre-chemotherapy samples, as well as a significant association between high WEE1 expression and poor prognosis in post-chemotherapy patients (Slipicevic et al. 2014). Furthermore, high-throughput screening of related kinases in ovarian cancer cells revealed that WEE1 kinase potentially regulated resistance to CHK1 inhibitors (Carrassa et al. 2012). Besides, WEE1 inhibition effectively reversed resistance to BRAF inhibitors (Haarberg et al. 2013; Sharma et al. 2013) and AURKA inhibitor (MLN8237) in HPV- HNSCC (head and neck squamous cell carcinoma (Lee et al. 2019). These findings indicate that WEE1 kinase may mediate the resistance to traditional chemotherapy and targeted therapy, making it a potential biomarker and therapeutic target for post-treatment resistance.

## Clinical development of WEE1 inhibitors

Despite considerable effort to develop small molecule inhibitors targeting WEE1 kinase, the available options are currently limited. Hitherto, a total of 79 clinical trials involving five WEE1 inhibitors have been reported by ClinicalTrials.gov (Table 1). Among these inhibitors, adavosertib (AZD1775) is the first-in-class potent inhibitor of WEE1 and has been involved in 61 trials. Following AZD1775, ZN-c3 has been studied in 12 trials, Debio-0123 in 4 trials, and SY-4835 and IMP7068 in only 1 trial each (Table 1). A sum of 28 clinical trials have been completed, with 9 trials dedicated to singular treatment and 19 trials investigating the efficacy of combination treatments.

**Table 1:** Small molecule inhibitors of Wee1 under clinical trials

Inhibitor	Properties	WEE1 IC50 (nM)*	Chemical structure*	Sponsor	Phases	Completed (n)/total (n)
adavosertib	A first-in-class, potent, and ATP-competitive specific small-molecule Wee1 inhibitor	5.2		AstraZeneca	II	28/61
ZN-c3	A novel, selective, and orally active bioavailable Wee1 protein kinase inhibitor	3.9		Zentalis Pharmaceuticals Inc	II	0/12
Debio-0123	An oral, potent, and highly selective Wee1 inhibitor	Nd		Almac Discovery Ltd	I	0/4
IMP7068	A potent, highly selective Wee1 inhibitor	Nd		Impact Therapeutics Inc	I	0/1
SY-4835	A new type of highly active and selective Wee1 small molecular inhibitor	Nd	Nd	Shouyao Holdings	I	0/1

*Nd not disclosed

The trials employing AZD1775 have yielded encouraging outcomes in prolonging progression-free survival of colorectal cancer patients with *RAS/TP53* mutations (Seligmann et al. 2021). Furthermore, a clinical trial conducted on human glioblastoma showed that AZD1775 successfully penetrated the blood–brain barrier, with great potential as a standalone therapeutic intervention for this disease (Sanai et al. 2018). In addition, AZD1775 has been extensively investigated as a means to enhance the efficacy of DNA damaging chemotherapy or radiation. For example, for refractory high-grade serous ovarian cancer, the combination of AZD1775 with gemcitabine has exhibited more significant therapeutic effect compared to gemcitabine alone (Lheureux et al. 2021). Similarly, the combination of AZD1775 with gemcitabine plus radiotherapy has been shown to improve survival in locally advanced pancreatic cancer (Cuneo et al. 2019). Furthermore, patients with platinum-sensitive *TP53*-mutant ovarian cancer benefited from the combination of AZD1775 with paclitaxel and carboplatin (Oza et al. 2020). These clinical trials offer important evidence advocating AZD1775 either as a monotherapy or in conjunction with other chemotherapeutic agents.

However, its application has been limited due to the associated toxicities. The most frequently observed toxicities included myelosuppression (such as anemia, neutropenia, and thrombocytopenia) and diarrhea (Do et al. 2015). In a separate investigation afflicted with advanced solid tumors, patients received the most prevalent drug-related adverse events (AEs) being diarrhea and fatigue when AZD1775 was administered as a sole monotherapy dose, while 19% of patients experienced severe treatment-related AEs, including fatigue, nausea, vomiting, diarrhea, anemia, neutropenia, and thrombocytopenia in conjunction of AZD1775 with various chemotherapeutic agents (gemcitabine, carboplatin, or cisplatin) (Leijen et al. 2016a). In the meantime, the safety, tolerability, and antitumor efficacy of combining AZD1775 with cisplatin and docetaxel in advanced HNSCC has been demonstrated (Méndez et al. 2018), suggesting that more investigations are needed to better balance the efficacy and toxicity of AZD1775.

ZN-c3 is another selective inhibitor of WEE1 kinase with significant anti-proliferative efficacy in various cancer cell lines. It is currently under evaluation in several clinical trials of different cancers, including ovarian cancer, solid tumors, and osteosarcoma (Clinicaltrials.gov). Notably, ZN-c3 possesses the advantage of a high maximum tolerated dose, enabling it to achieve the similar growth/proliferation inhibition at significantly lower doses compared to AZD1775. Although ZN-c3 exhibits greater specificity compared to AZD1775, its inhibitory activity has been observed on multiple kinases, and common side effects such as fatigue, vomiting, diarrhea, and nausea have been consistently reported in several studies (Li et al. 2021; Tolcher et al. 2021). Nevertheless, ZN-c3 has demonstrated its efficacy as a standalone treatment for cancer cells, displaying high tolerance and satisfactory safety profiles.

Conversely, the remaining three WEE1 inhibitors, namely Debio-0123, SY-4835, and IMP7068, are still under clinical evaluation. Thus, the efficacy and potential toxicity of these inhibitors have yet to be definitively established.

## Predictive biomarkers

**Fig. 2:**
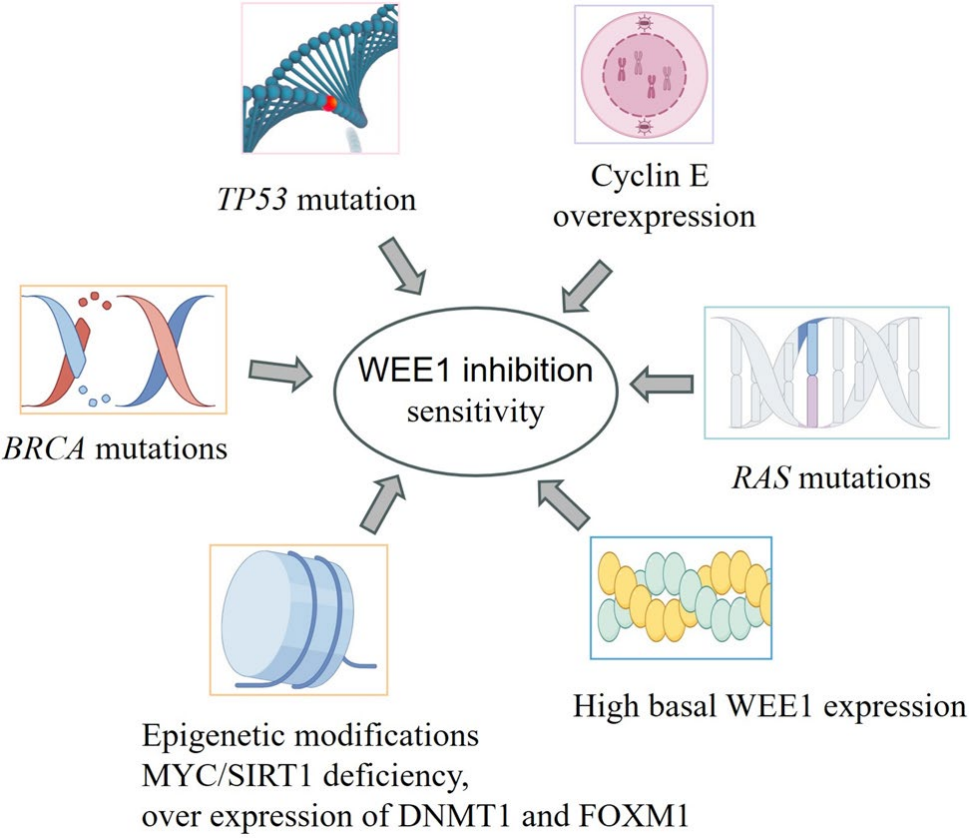
Biomarkers of sensitivity to WEE1 inhibition therapy

Despite the considerable therapeutic potential of WEE1 inhibition as standalone treatment or in combination therapies, biomarkers that accurately predict its susceptibility and resistance remains to be identified. Next, we summarize the potential biomarkers of WEE1 inhibitory treatment described so far (Fig. 2).

### Biomarkers of sensitivity to WEE1 inhibition therapy

#### *TP53* mutation

The correlation between *TP53* mutation status and the sensitivity to AZD1775 have been extensively established (Bauman and Chung 2014; Bridges et al. 2011; Diab et al. 2019; Hirai et al. 2009; Ku et al. 2017; Yang et al. 2020). In addition, AZD1775 selectively sensitized p53-deficient cancer cells to radiotherapy, gemcitabine, carboplatin and cisplatin, compared with the isogenic wild-type line (Bridges et al. 2011; Hirai et al. 2009). Consistently, cells with dysfunctional p53 exhibited heightened sensitivity to WEE1 inhibition when combined with conventional chemotherapy and ionizing radiation (Barbosa et al. 2019; Wang et al. 2001; Yin et al. 2018). These investigations propose that *TP53* mutation or dysfunction may serve as a bona fide biomarker for AZD1775 sensitivity.

#### High basal WEE1 expression

Gastric cancer cells exhibiting high expression of WEE1 displayed increased susceptibility to WEE1 inhibitory therapy (Kim et al. 2016), indicating that the efficacy of WEE1 inhibition may be dependent on the expression level of WEE1 kinase. Another study of 663 advanced non-small cell lung cancer patients showed that WEE1 rs3910384 genotype was markedly correlated with prognosis following platinum-based chemotherapy as well as the combined efficacy of platinum and gemcitabine (Liu et al. 2015). Furthermore, co-administration of AZD1775 and cisplatin demonstrated a synergistic effect in a patient-derived xenograft (PDX) model characterized by elevated basal expression of PAXIP1 and WEE1 (Jhuraney et al. 2016). These findings indicate that the basal level of WEE1 kinase may play a part in the responsiveness to WEE1 inhibition therapy.

#### *RAS* mutations

The particular status of *KRAS* mutation is critical to the response of non-small cell lung cancer (NSCLC) cells to sorafenib, a multi-target tyrosine kinase inhibitor. High-throughput screening utilizing a siRNA library targeting 719 human kinases identified WEE1 as a modulator of sorafenib response, while AZD1775 was observed to augment the susceptibility of *KRAS* mutated NSCLC cells towards sorafenib (Caiola et al. 2018). In addition, the concurrent inhibition of mTOR and WEE1 has been demonstrated to elicit robust synergistic cytotoxic effects in NSCLC cell lines harboring *KRAS* mutations. This combination therapy was also found to impede the growth of human tumor xenografts and induce tumor regression in a murine model of lung adenocarcinoma (Hai et al. 2017). Similarly, a notable observation was made regarding the combined effect of mTOR inhibitor and WEE1 inhibitor in both mutant neuroblastoma *NRAS*- and mutant *KRAS*-positive acute myelogenous leukemia (AML) cell lines and primary patient samples. It is worth mentioning that these findings have been shown to be applicable to other malignancies expressing mutant *RAS*, such as mutant *NRAS*-positive melanoma, and mutant *KRAS*-positive colorectal cancer, pancreatic cancer, and lung cancer (Weisberg et al. 2015). Moreover, combination of AZD1775 and cisplatin significantly prolonged overall survival in a genetically engineered mouse model of mutant *KRAS* with concomitant loss of *LKB1*. Of note, *LKB1* is among the frequently mutated genes in NSCLC and commonly co-occurs with *KRAS* mutations (Richer et al. 2017). These findings suggest that WEE1 inhibitors exhibit promising anti-tumor potential when used in conjunction with other small molecule inhibitors in the context of *RAS* mutations.

#### *BRCA* mutations

A previous Phase I Study (NCT01748825) evaluated the single-agent activity of AZD1775 in patients with BRCA mutations. Notably, two patients with BRCA mutations, one with head and neck cancer and one with ovarian cancer, exhibited partial response (Do et al. 2015). Chen et al. demonstrate, using various in vitro and in vivo model systems, that triple-negative breast cancers (TNBCs) with either BRCA1/2 mutations or cyclin E overexpression exhibit heightened susceptibility to AZD-1775 when administered in conjunction with MK-4837 (a PARP inhibitor). The combined treatment of these two agents led to synergistic eradication of TNBC cells, which was attributed to the induction of replicative stress, downregulation of DNA repair mechanisms, failure in cytokinesis, and ultimately increased apoptosis (Chen et al. 2021). These findings highlight the potential clinical application of using BRCA mutations as biomarkers to select patients that may benefit from therapies involving AZD1775.

#### Cyclin E overexpression

Recently, cyclin E levels have been shown to correlate with the efficacy of AZD1775 in breast cancer models (Chen et al. 2018). Chen et al. conducted a study wherein they found that overexpression of cyclin E is more prevalent in TNBCs exhibiting high rates of recurrence and these cells more susceptible to inhibition of wee1 kinase. Furthermore, they also discovered that the overexpression of Cyclin E induces the activation of DNA replication stress pathways in a CDK2-dependent manner, consequently augmenting the activity of Wee1 kinase (Chen et al. 2018). Their study suggests that Cyclin E overexpression may serve as a biomarker of sensitivity to WEE1 inhibitors.

#### Epigenetic modifications

Interestingly, small cell lung cancers with alterations in the MYC family exhibited increased sensitivity to combined treatment of Olaparib and AZD1775 (Lallo et al. 2018). Conversely, AZD1775 enhanced gemcitabine sensitivity despite the presence of elevated c-MYC expression in medulloblastoma (Moreira et al. 2020). Our previous study indicated that tumor cells exhibiting elevated basal levels of DNMT1 and FOXM1 displayed heightened sensitivity to AZD1775 (Guo et al. 2022). In addition, the latest research reports that sirtuin 1 (SIRT1) deficiency induces WEE1 hyperacetylation and activation, rendering cancer cells resistant to WEE1 inhibition (Zhu et al. 2023). These findings imply that epigenetic alterations may play a role in the responsiveness of WEE1 inhibitory therapy.

### Biomarkers associated with resistance to WEE1 inhibition

**Fig. 3:**
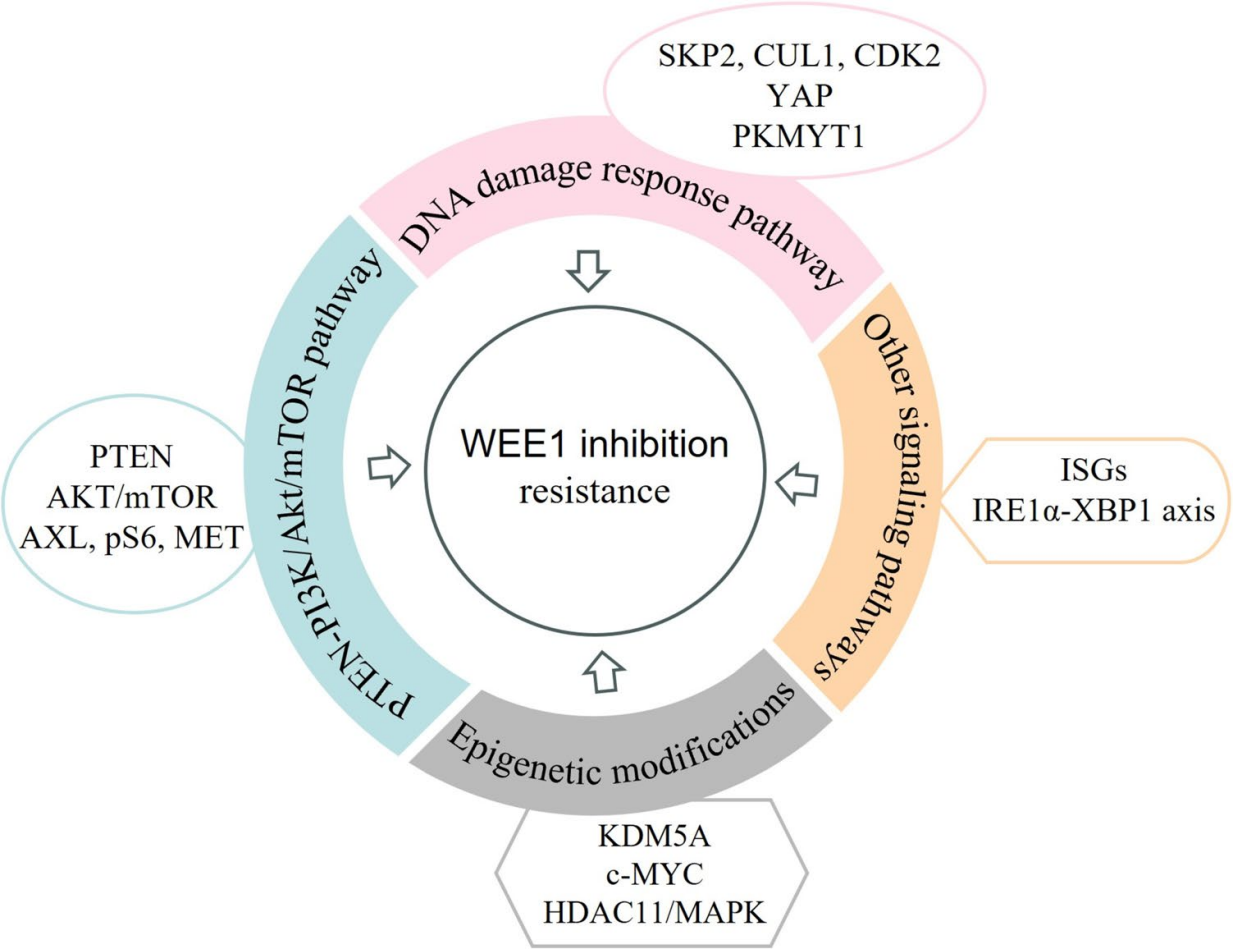
Biomarkers of resistance to WEE1 inhibition therapy

Despite numerous studies demonstrating the considerable effectiveness of WEE1 inhibition therapy in combating tumors, the emergence of primary and secondary resistance remains an inevitable challenge. Therefore, a summary of biomarkers predicting resistance to WEE1 inhibitors will help formulate treatment strategies to avoid it (Fig. 3).

#### Biomarkers in the PTEN-PI3K/Akt/mTOR signaling pathway

It has been recently reported that breast cancer cells with high PTEN expression can regain their activity after discontinuation of AZD1775 treatment (Brunner et al. 2020). This suggests that the presence of high levels of PTEN may contribute to resistance against WEE1 inhibitors. Furthermore, a high-throughput proteomic analysis revealed the overexpression of AKT/mTOR pathway molecules and phosphorylated S6 ribosomal protein in small cell lung cancer and ovarian cancer models that exhibit primary resistance to AZD1775 (Li et al. 2020a; Sen et al. 2017). Similarly, our previous high-throughput Reverse Phase Protein Array (RPPA) observed a compensatory up-regulation of the mTOR pathway following AZD1775 treatment during early stages of ovarian cancer. Combination of WEE1 and mTOR dual inhibition demonstrated synergistic effects in both ovarian cancer cell lines and PDX models (Li et al. 2020a). These findings suggest that activation of the PTEN-PI3K/Akt/mTOR signaling pathway may play a crucial role in conferring resistance to WEE1 inhibition therapy.

#### Biomarkers in DNA damage response pathway

In a comprehensive investigation involving ovarian cancer, chronic myeloid leukemia, and breast cancer, the surviving cells following WEE1 inhibitor treatment were subjected to sequencing analysis. The results revealed that G1/S regulatory genes (SKP2, CUL1, CDK2) were significantly enriched in the surviving cells, where inhibiting these genes effectively mitigated the formation of DNA damage (Heijink et al. 2015). Additionally, another study in ovarian cancer showed that the resistance to AZD1775 therapy was mediated by YAP through the E2F1 DNA damage response axis (Oku et al. 2018). Notably, independent studies consistently demonstrated a correlation between high expression of PKMYT1 and reduced sensitivity to AZD1775 (Ghelli Luserna Di Rorà et al. 2018; Lewis et al. 2019). These findings propose that the resistance to WEE1 repression may be attributed to adaptive responses within the DNA damage response pathway.

#### Biomarkers in epigenetic modifications

It was recently shown that the survival of AZD1775-resistant acute leukemia cell lines relied on the activity of MYC and HDAC, which was in part due to the increased KDM5A activity (Garcia et al. 2020), thus corroborating preclinical studies that advocate for the combined utilization of WEE1 and HDAC inhibitors (Qi et al. 2015; Tanaka et al. 2017; Zhou et al. 2015). Similarly, research by Zhou et al. also found that the WEE1 inhibitor PD0166285 can arise the expression of HDAC11 which was negatively correlated with survival of AML patients. Mechanistically, HDAC11 can reduce the anti-tumor effect of PD0166285 through an effect on p53 stability and the changes in phosphorylation levels of MAPK pathways (Zhou et al. 2023). The above studies suggest that epigenetic modifications, such as HDACs, are involved in mediating resistance to WEE1 inhibitor treatment, and targeting these molecules can effectively reverse WEE1 treatment resistance.

#### Biomarkers in other signaling pathways

Furthermore, our recent study revealed that ovarian and colorectal cancer cells sensitive to AZD1775 monotherapy exhibited an up-regulation of interferon signaling gene groups in a responsive manner (Guo et al. 2022). This finding was further confirmed through RNA-seq analysis of murine AZD1775-resistant ovarian cancer cells (Guo et al. 2022), suggesting that adaptive immune signaling may play a role in the development of resistance to AZD1775. In keeping with this, we also demonstrated that refractory ovarian cells can enhance the ability to adapt to AZD1775 treatment by activating the IRE1α-XBP1 axis of the unfolded protein response pathway (Xiao et al. 2022). Our study suggests that immune and unfolded protein response may be involved in WEE1 therapeutic resistance.

## Conclusion

WEE1 inhibition has exhibited significant potential in tumor treatment. Preclinical investigations have paved the way for the advancement of clinical trials involving WEE1 inhibitors. The refinement of predictive biomarkers would help identifying populations that would benefit from WEE1 inhibitor therapy. Understanding the prognostic significance of these biomarkers in relation to tumor treatment outcomes and patient prognosis is key to the development of appropriate treatment strategy. Despite these important progress, further investigation is warranted to identify additional biomarkers that can inform the development of treatment strategies.

## Data Availability

All data generated or analyzed during this study are included in this published article.
